# Impact of Concomitant Use of Proton Pump Inhibitors and Clopidogrel on Recurrent Stroke and Myocardial Infarction

**DOI:** 10.3390/ph16091213

**Published:** 2023-08-28

**Authors:** Yong Kang Lee, Hyun Sun Lim, Youn I Choi, Eun Ju Choe, Seonji Kim, Seng Chan You, Kyung Joo Lee, Yerim Kim, Da Hee Park, Woon Geon Shin, Seung In Seo

**Affiliations:** 1 Department of Internal Medicine, National Health Insurance Service Ilsan Hospital, Goyang 10444, Republic of Korea; lyk8266@gmail.com (Y.K.L.); cys7like@nhimc.or.kr (Y.I.C.); choeej15@gmail.com (E.J.C.); 2 Department of Research and Analysis, National Health Insurance Service Ilsan Hospital, Goyang 10444, Republic of Korea; hsunlim@nhimc.or.kr; 3 Department of Biomedical Systems Informatics, Yonsei University College of Medicine, Seoul 03722, Republic of Korea; seonji0316@yuhs.ac (S.K.); applegna@gmail.com (S.C.Y.); 4 Institute for Innovation in Digital Healthcare, Yonsei University, Seoul 03722, Republic of Korea; 5 Department of Medical Informatics & Statistics, Kangdong Sacred Heart Hospital, Seoul 05355, Republic of Korea; 226002@kdh.or.kr; 6 Department of Neurology, Kangdong Sacred Heart Hospital, Hallym University College of Medicine, Seoul 24252, Republic of Korea; brainyrk@kdh.or.kr; 7 Institute for Liver and Digestive Diseases, Hallym University, Chuncheon 24252, Republic of Korea; dahee0809@kdh.or.kr (D.H.P.); sgun9139@gmail.com (W.G.S.); 8 Department of Internal Medicine, Kangdong Sacred Heart Hospital, Hallym University College of Medicine, Seoul 05355, Republic of Korea

**Keywords:** proton pump inhibitors, clopidogrel, stroke, myocardial infarction

## Abstract

Background/Aims: Conflicting results have been reported regarding the interaction between proton pump inhibitors (PPIs) and clopidogrel. We investigated whether concomitant PPI use influenced the risk of recurrence in patients with stroke and myocardial infarction (MI). Methods: This study used two databases for two different designs, the Korean National Health Insurance Service (NHIS) database for a self-controlled case series design, and the national sample cohort of the NHIS data base converted to the Observational Medical Outcomes Partnership-Common Data Model version for a cohort study based on large-scale propensity score matching. Results: In the PPI co-prescription group, recurrent hospitalization with stroke occurred in 17.6% of the 8201 patients with history of stroke, and recurrent MI occurred in 17.1% of the 1216 patients with history of MI within1 year. According to the self-controlled case series, the overall relative risk (RR) of recurrent stroke was 2.09 (95% confidence interval (CI); 1.83–2.38); the RR showed an increasing trend parallel to the time from the beginning of PPI co-prescription. In the cohort study, there was a higher incidence of recurrent stroke in the PPI co-prescription group (Hazard ratio (HR): 1.34, 95% CI: 1.01–1.76, *p* = 0.04). The overall RR of recurrent MI was 1.47 (95% CI; 1.02–2.11) in the self-controlled case series; however, there was no statistically significant difference in recurrent MI in the cohort study (HR:1.42, 95% CI:0.79–2.49, *p* = 0.23). The impact of individual PPIs on stroke and MI showed different patterns. Conclusions: A PPI co-prescription >4 weeks with clopidogrel was associated with hospitalization of recurrent stroke within 1 year of initial diagnosis; however, its association with recurrent MI remains inconclusive. The influence of individual PPIs should be clarified in the future.

## 1. Introduction

Proton pump inhibitors (PPIs) promote peptic ulcer recovery by suppressing gastric acid secretion and reduce the incidence of complications from ulcers such as perforation or bleeding [1]. PPIs improve reflux symptoms and prevent complications from reflux esophagitis [2,3,4] and are fundamental in treating gastroesophageal reflux disease. PPIs also prevent gastrointestinal bleeding in patients using antithrombotic agents or non-steroidal anti-inflammatory drugs [5] and have, therefore, been the basis for the treatment of various gastrointestinal diseases.

As PPI use increases worldwide, growing concerns about PPI complications have been raised [6,7,8]. Specifically, drug interaction with clopidogrel, which is an antiplatelet agent classified as thienopyridines, has been postulated in many recent studies [9,10,11,12,13,14,15,16,17,18,19,20,21]. Both PPIs and clopidogrel are metabolized by hepatic cytochrome P450 (CYP) enzymes; in the presence of CYP2C19 inhibition, PPIs could reduce the efficacy of clopidogrel’s protective roles in cardiovascular events [22]. Moreover, when clopidogrel and PPIs were co-administered, the ability of platelet aggregation increased and the results supported the hypothesis that the concentration of the metabolite of clopidogrel would be lowered [23,24]. Hence, The US Food and Drug Administration (FDA) issued safety announcements between January 2009 and October 2010 warning against the concomitant use of clopidogrel and PPIs, especially omeprazole and esomeprazole, due to a potential drug interaction that may attenuate clopidogrel’s antiplatelet activity [25]. However, despite the FDA warnings, the real-world evidence of clopidogrel and individual PPI interaction has not been fully evaluated, especially in Asian populations which are known to havea high frequency of poor metabolizer CYP2C19 enzymes. In addition, previous observational studies were limited by multiple confounding factors and small sample sizes.

To overcome these limitations, we conducted a nationwide population-based study using two different designs: a self-controlled case series and a large-scale propensity score (PS) matching. We aimed to identify the risk of recurrent events in patients diagnosed with stroke and myocardial infarction (MI) and the concomitant use of PPIs and clopidogrel. We also evaluated the impact of the concomitant use of individual PPIs and clopidogrel on recurrent stroke and MI.

## 2. Results

### 2.1. Self-Controlled Case Series Analysis

The comparison of the baseline characteristics between the PPI co-prescription and non-prescription groups for stroke and MI is presented in Table 1. The PPI co-prescription group was significantly older than the PPI non-prescription group. There was a significant difference in the proportion of comorbidities between the two groups (Table 1). In the group of patients diagnosed with stroke, the PPI co-prescription group had a higher proportion of women (45.8% vs. 41.4%) and older age (67.3 vs. 66.1 years). There were more patients with hypertension, dyslipidemia, and never-smokers in the PPI co-prescription group; diabetes was seen less in the PPI co-prescription group than the clopidogrel monotherapy group. There was no difference in BMI between the two groups. The total cholesterol level was lower in the PPI co-prescription group (202.52 mg/dL vs. 204.96 mg/dL) (Table 1).

In the group of patients diagnosed with MI, the PPI co-prescription group had a lower proportion of women (55.1% vs. 64.0%) and older age (69.6 vs. 68.1 years). Hypertension and never smokers were more prevalent (58.1% vs. 86.4% and 28.0 vs. 24.1%) in the PPI co-prescription group than the clopidogrel monotherapy group. There was no difference in the prevalence of diabetes and dyslipidemia between the two groups. BMI was lower in PPI co-prescription group (24.11 vs. 24.41). Considering the differences between the PPI co-prescription group and nonprescription group, we performed a self-controlled case series analysis to overcome confounding factors between individuals. Of those, we included 1448 hospitalizations with recurrent stroke and 208 hospitalizations with recurrent MI in the PPI co-prescription group.

#### 2.1.1. Relative Risk of Recurrent Stroke in the PPI Co-Prescription Group

Of the 1448 hospitalizations with recurrent stroke as the primary diagnosis, 653 (45.1%) events occurred while patients were co-prescribed PPIs and 795 (54.9%) events occurred while patients were prescribed clopidogrel alone. The overall relative risk of recurrent stroke was 2.09 (95% CI; 1.83–2.38) (Table 2). According to the time from the beginning of PPI co-prescription, the RR showed an increasing trend (0~2 weeks; 1.76 (95% CI; 1.50–2.07), 2~4 weeks; 2.02 (95% CI; 1.68–2.43), 4~6 weeks; 3.02 (95% CI; 2.36–3.86), 6~8 weeks; 2.81 (95% CI; 2.02–3.92), >8 weeks; 5.57 (95% CI; 4.06–7.64))(Table 2). We also conducted an analysis including PPI washout periods. During the remaining 4 weeks after the end of the PPI prescription, 201 events occurred. The RR including the 4week washout periods was 2.47 (95% CI 2.16–2.80) (Table 2).

To identify the impact of individual PPIs on clopidogrel, we repeated the analysis according to PPI type. The most-prescribed drug was rabeprazole (*n* = 492), followed by esomeprazole (*n* = 373), pantoprazole (*n* = 364), lansoprazole (*n* = 170), omeprazole (*n* = 137), and dexlansoprazole (*n* = 12) in patients with recurrent stroke. Table 3 shows the RR of risk periods during PPI co-prescription periods only, including the 4week PPI washout periods. In the analysis of stroke, it showed a significant risk in the overall PPI exposed periods including omeprazole, esomeprazole, pantoprazole, and rabeprazole. In addition, esomeprazole showed the highest RR (2.75. 95% CI; 2.12–3.57) and the risk was more significant, including during the washout period (Table 3). In the analysis of recurrent MI, only pantoprazole showed a significant RR (2.56, 95% CI; 1.46–4.50).

#### 2.1.2. Relative Risk of Recurrent MI in the PPI Co-Prescription Group

Of the 208 hospitalizations with recurrent MI as the primary diagnosis, 95 events (45.7%) occurred while patients were co-prescribed PPIs and 113 events (54.3%) occurred while patients were prescribed clopidogrel alone. The overall relative risk of recurrent MI was 1.47 (95% CI; 1.02–2.11) (Table 2). According to PPI co-prescription duration, the RR showed an increasing trend (0~2 weeks; 1.30 (95% CI; 0.83–2.04), 2~4 weeks; 0.95 (95% CI; 0.54–1.69), 4~6 weeks; 2.33 (95% CI; 1.32–4.13), 6~8 weeks; 1.99 (95% CI; 0.98–4.03), >8 weeks; 3.80 (95% CI; 1.93–7.45))(Table 2). We also conducted an analysis including the PPI washout periods, and the RR including the 4week washout periods was 1.87 (95% CI:1.31–2.65) (Table 2).

The most commonly prescribed drug was pantoprazole (*n* = 82), followed by rabeprazole (*n* = 58), esomeprazole (*n* = 50), lansoprazole (*n* = 32), omeprazole (*n* = 9), and dexlansoprazole (*n* = 2). Only pantoprazole showed a significant RR for MI (2.56, 95% CI; 1.46–4.50) (Table 3).

### 2.2. Cohort Study

The cohort study was based on the NHIS-CDM database and the study flow chart of the cohort study is shown in Figure S1. Initially, 442 patients in the PPI co-prescription group and 11,078 patients in the non-prescription group were included in the analysis of stroke, and 245 patients in the PPI co-prescription group and 3933 patients in the non-prescription groups were included in the analysis of MI. A total of 9947 and 7979 covariates were used for large-scale PS matching in the analysis of stroke and MI, respectively (Figure S2). The baseline characteristics of stroke and MI before and after 1:4 PS matching are presented in Supplementary Tables S1 and S2, respectively. After PS matching, 373 patients were included in the PPI co-prescription group, 1051 in the non-prescription group, 179 in the PPI co-prescription group, and 439 in the non-prescription groups in the final analysis of stroke and MI, respectively. The most standardized mean difference was less than 0.1, which suggests that the PPI co-prescription and non-prescription groups were well balanced after large-scale PS matching.

#### 2.2.1. Incidence of Recurrent Stroke in the PPI Co-Prescription Group

The results of a Cox regression analysis with 1:4 PS matching are shown in Table 4. There was a higher incidence of recurrent stroke in the PPI co-prescription group (PPI co-prescription group (*n* = 373) vs. non-prescription group (*n* = 1051); 81/240 person years vs. 189/740 person years, HR: 1.34, 95% CI; 1.01–1.76, *p* = 0.04). A sensitivity analysis was performed using 1:1 matching, PS stratification, and different observation periods. In the analysis of stroke, the sensitivity analysis showed results consistent with those of the main analysis (Table 5).

#### 2.2.2. Incidence of Recurrent MI in the PPI Co-Prescription Group

There was no statistically significant difference in the incidence rate of recurrent MI between the PPI co-prescription and non-prescription groups (PPI co-prescription group (*n* = 179) vs. non-prescription group (*n* = 439); 23/133 person years vs. 43/336 person years, HR:1.42, 95% CI: 0.79–2.49, *p* = 0.23) (Table 4). A sensitivity analysis also showed inconsistent results (Table 5). Only the results with 1:4 PS matching until 6 months showed a significantly higher incidence of recurrent MI in the PPI co-prescription group (HR:2.15; 95% CI:1.10–4.13).

## 3. Discussion

In the present study, the risk of recurrent stroke was higher in the PPI co-prescription group with clopidogrel in patients with stroke in both the self-controlled case series analysis and the large-scale PS-matched cohort study. However, the risk of recurrent MI showed different results among various analyses, suggesting that the interaction between PPI and clopidogrel on MI should be elucidated. In addition, we evaluated the impact of the concomitant use of individual PPIs and clopidogrel on cardiovascular event rates. To date, there have been few large-scale Asian studies on the interaction of PPI and clopidogrel on cardiovascular outcomes by using various study designs, including the analysis of individual PPIs at the population level.

Our study results are consistent with a recent meta-analysis including 22 studies [14]. The study revealed that concomitant use of PPI with thienopyridines was associated with an increased risk of stroke (hazard ratios adjusted, 1.30; 95% CI, 1.04–1.61; *p* = 0.02), composite stroke/MI/cardiovascular death (hazard ratios adjusted, 1.23; 95% CI, 1.03–1.47; *p* = 0.02), but not with MI (hazard ratios adjusted, 1.19; 95% CI, 0.93–1.52; *p* = 0.16) [14]. In the UK General Practice Research Database cohort study, the hazard ratio for the association between PPI use and death or MI incidence was 1.37 (95% CI, 1.27–1.48), but there was a lack of specific association in the self-controlled case series analysis [17]. On the contrary, meta-analyses of studies published in 2012–2016 showed that the combined use of clopidogrel with PPI is associated with significantly higher adverse cardiovascular events, such as major adverse cardiac events (MACE), stent thrombosis, and MI following PCI [26]. However, most of these studies were Western observational studies and the types of PPI were distributed heterogeneously [26].

Notably, CYP2C19 is subject to genotypic variation, with some individuals and ethnicities having naturally poor 2C19 metabolic activity. CYP2C19 poor metabolic phenotypes are found in 13–23% of healthy East Asian populations but in only 2–5% of Caucasians [12]. A recent meta-analysis evaluated the effect of the concomitant use of dual antiplatelet therapy and PPI and assessed the effect of ethnic variance on clinical outcomes [27]. The PPI co-medication was associated with increased risk for all endpoints among Caucasian populations; however, there was no association with increased risk for MACE, all-cause death, and cardiac death among Asian populations [27]. In another 12,440 multi-ethnic Asian population study, the risk of subsequent MI was higher in the Malay and Chinese populations than in the Indian population [9]. Our study was the first large-scale observational study of the interaction between PPI and clopidogrel on cardiovascular outcomes in the Korean population.

PPIs are metabolized mainly by CYP2C19 and the inhibition of this enzyme is heterogeneous within the class of PPIs. PPIs are classified based on their binding affinity for CYP2C19, including those with high and low CYP2C19inhibitory potential [23]. After the FDA’s warnings against the use of clopidogrel with inhibiting PPIs (omeprazole and esomeprazole), treatment with inhibiting PPIs and clopidogrel has continued to decrease since 2010 [28]. To date, however, there have been few studies comparing the impact of individual PPIs with clopidogrel on cardiovascular outcomes, and conflicting results have been reported [29,30]. A previous Korean randomized controlled trial revealed that pantoprazole does not increase platelet aggregation in patients receiving dual antiplatelet therapy [30]. However, a recent meta-analysis showed that post-PCI patients on dual antiplatelet therapy with clopidogrel in the PPI group were associated with a higher risk of MACE and MI in pantoprazole, lansoprazole, omeprazole, and esomeprazole, except for rabeprazole, although the results were based on a small number of studies [29]. In our study, the impact of individual PPIs on stroke and MI showed different patterns. Esomeprazole had the highest RR, followed by rabeprazole, omeprazole, and pantoprazole in recurrent stroke. Lansoprazole and dexlansoprazole were not significantly associated with recurrent strokes. In contrast, only pantoprazole showed a significant risk of recurrent MI. However, our results should be interpreted with caution because the sample size of individual PPIs was relatively small in the analysis of recurrent MI. Therefore, it may be necessary to directly compare the impact of individual PPIs on clopidogrel in the future.

The main advantage of this study was the use of two different designs. To overcome the weakness of cohort study based on traditional PS adjustment (with manually selected covariates), we performed a cohort study based on large-scale PS matching and a self-controlled case series design with two different databases. The primary study design was a self-controlled case series in the Korean NHIS database. The advantage of the design is that it is self-controlled; it implicitly adjusts for all confounders that remain fixed over the observation period, such as genetic and socio-economic factors [31]. Thus, the design removes all fixed confounding between individuals as the comparisons are made within an individual, relying on patients who have both exposed and unexposed periods for the main comparison, which was also applied to the previous study on PPI and clopidogrel [17]. We also performed a self-controlled case series analysis according to the time interval from the beginning of PPI co-prescription to show the dose–response relationship, including PPI washout periods. The secondary study design was a large-scale PS matching-based cohort study using the OMOP-CDM-based database. In the traditional cohort study, at baseline, PPI users tended to be older and have more comorbidities than non-users, despite adjusting for known confounding factors. Our study also showed significantly more comorbidities in the baseline PPI co-prescription group. The large-scale PS matching included all previously recorded comorbidities, all drugs, and Charlson comorbidity index scores before cohort entry as covariates, which enabled the adjustment of unmeasured confounding factors [32,33]. Recently, Zhang et al. demonstrated that a large-scale PS model may adjust for indirectly measured confounders by including tens of thousands of covariates that may be correlated with them [32]. In addition, multiple sensitivity analyses with different matching ratios and methods showed consistent results in the analysis of stroke; however, inconsistent results were obtained in the analysis of MI. The OMOP-CDM-based research also has the strength to be applied to other ethnic groups through the common analytic R code [34,35].

Despite the strengths, the present study had several limitations. First, we defined the recurrence of stroke or MI as hospitalization with a primary diagnostic code, which might have been overestimated. Moreover, the recurrence rates of stroke and MI in this study were higher than those reported in previous studies. Second, in the self-controlled case series, the event should not condition the probability of subsequent exposure [36]. To ensure this assumption, a “pre-exposure” time risk window can be created to examine whether the exposure depends on the occurrence of the outcome [36]. We could not establish a pre-exposure time period; therefore, there could be potential bias in the self-controlled case series design. Third, large-scale PS matching leads to inevitable case loss; thus, it might have influenced the statistically insignificant result in the analysis of MI. We also could not separate individual PPIs in the cohort study due to the small sample size. Finally, despite efforts to reduce confounding factors, the current study was an observational study. The possibility of misclassification or residual biases exists because of the limitations of the claim database.

## 4. Materials and Methods

### 4.1. Data Sources

This study utilized two databases: (1) Korean National Health Insurance Service (NHIS) database from 2009 to 2014 for self-controlled case series study and (2) Observational Medical Outcomes Partnership-Common Data Model (OMOP-CDM) version of National Sample Cohort (NSC) from the NHIS database between 2002 and 2013 for cohort study. The Korean NHIS database is a large dataset of 1.3 trillion records, including diagnoses, procedures, surgeries, and prescriptions. It also contains medical data, including sociodemographic status, body height and weight, comorbidities, and health-related behaviors such as alcohol consumption and smoking habits [37,38]. In the OMOP-CDM version of the Korean NHIS-NSC (NHIS-CDM), the data of 1.13 million patients were converted to the OMOP-CDM, resulting in a 99.1% conversion rate [39]. The OMOP-CDM represents healthcare data from a diverse source in a consistent and standardized manner [40]. Furthermore, the NHIS-CDM database has been applied in multiple studies [41,42,43]. The study protocol was approved by the Institutional Review Board of Kangdong Sacred Heart Hospital (IRB number: 2021-07-011) and National Health Insurance Service Ilsan Hospital (IRB number: 2020-06-037).

### 4.2. Study Design and Population

#### 4.2.1. Self-Controlled Case Series Analysis

**Figure 1:**
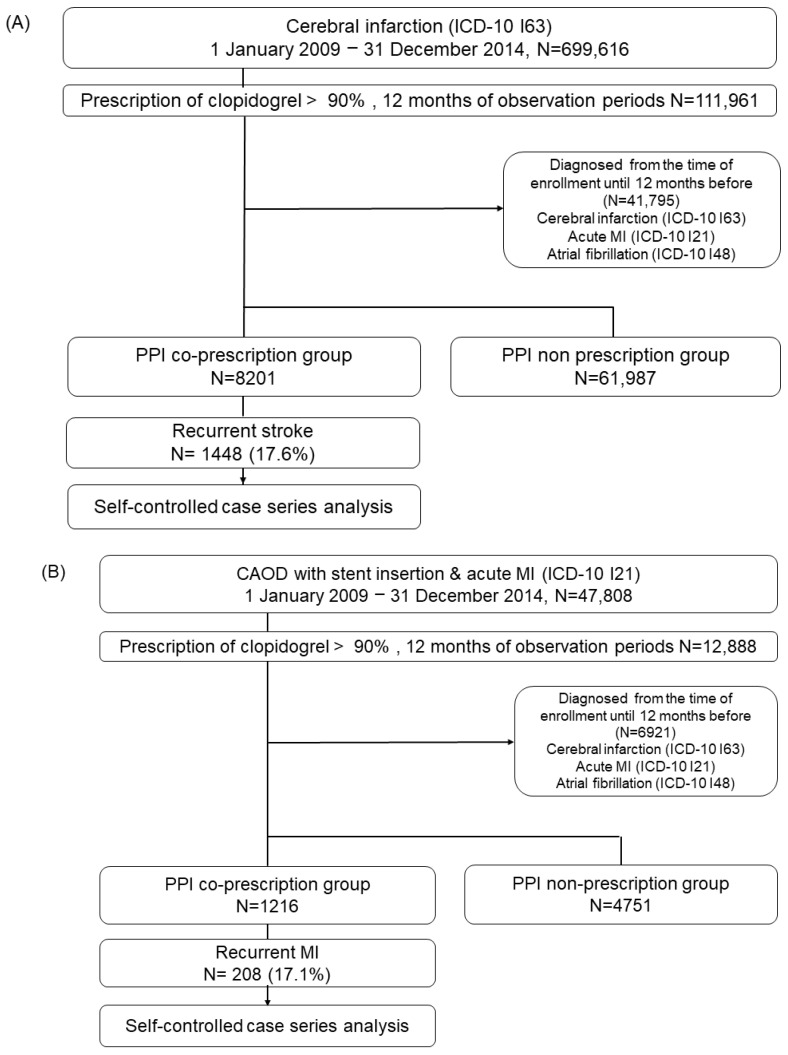
Study flow chart in the self-controlled case series of recurrent stroke (**A**) and myocardial infarction (**B**) in the Korean NHIS database. PPI, proton pump inhibitor; MI, myocardial infarction; ICD, International Classification of Diseases; CAOD, coronary artery obstructive disease.

We included adult patients from the Korean NHIS database who were newly diagnosed with cerebral infarction (ICD-10 I63) and acute MI (ICD-10 I21) or percutaneous coronary intervention from 1 January 2009 to 31 December 2014 and prescribed clopidogrel during >90% of the observation period. The PPI co-prescription group was defined as PPI prescription >4 weeks. The exclusion criteria were as follows: (1) observation period <1 year before cohort entry, (2) atrial fibrillation, and (3) PPI exposure <4 weeks. The flow of the study is presented in Figure 1. In the PPI co-prescription group, recurrent hospitalization due to stroke occurred in 17.6% of the 8201 patients with a history of stroke (Figure 1A), and recurrent MI occurred in 17.1% of the 1216 patients with a history of MI within 1 year after the initial diagnosis (Figure 1B).

Self-controlled case series analysis is a type of cohort study in which relative risk is based on within-person comparisons between exposed and unexposed observation times, meaning that only exposed patients with events can be included [31]. We included only patients with recurrent stroke (*n* = 1448) and MI (*n* = 208) in the PPI co-prescription group for the self-controlled case series analysis. We considered PPI exposure to begin on the date of PPI prescription and end after its calculated duration, including any consecutive prescriptions, plus an additional 30-day washout period. The time interval from beginning of PPI co-prescription was divided as follows: 0–2, 2–4, 4–6, 6–8, and >8 weeks from PPI start. We also analyzed the overall relative risk for each PPI type.

#### 4.2.2. Cohort Study

The cohort study was conducted using the NHIS-CDM database, which could be analyzed with large-scale PS matching to improve comparability between PPI and non-PPI groups. The target cohort was defined as new clopidogrel use over 4 weeks with a co-prescription of PPI over 4 weeks in patients diagnosed with stroke or acute MI. The target cohort was censored if patients were diagnosed with any of the outcomes or if the observation ended. The comparator cohort was defined as new clopidogrel use over 4 weeks with no PPI exposure within 4 weeks of clopidogrel exposure in patients with stroke or acute MI. The censoring rule was PPI exposure, outcome ascertainment, or end of observation. The observation period before cohort entry was at least 1 year for both cohorts. The primary outcome was the incidence rates of recurrent stroke or MI with hospitalization in patients with a history of stroke or MI, respectively.

### 4.3. Statistical Analyses

We used SAS version 9.4 (SAS Institute, Cary, NC, USA) and Stata version 15.1 (Stata Corporation, College Station, TX, USA) for all statistical analyses in the self-controlled case series. Categorical variables were analyzed using the chi-squared test, and continuous variables were analyzed using *t*-tests. We calculated the incidence rate ratio, which is the ratio of the incidence rate of an event during the exposure period to the non-exposure period and described the relative risk and 95% confidence interval (CI).

The cohort study was performed using ATLAS ver. 2.7 and R statistical software (version 3.6.1 for Windows; R Foundation for Statistical Computing). We analyzed Cox proportional hazard models to calculate the hazard ratios (HRs) with 95% CI for outcomes between the target and comparator cohorts using the CohortMethod package in R. The Kaplan–Meier method was used to estimate the cumulative incidence rates, and the cumulative incidence between the two groups was compared using the log-rank test.

The OMOP-CDM research provides large-scale PS models with regularized logistic regression [32,33]. The following covariates were used for PS matching: age, sex, all previous comorbidities, all drugs in the 365 days prior to the index date, and the Charlson comorbidity index score. We considered the main analysis to be 1:4 PS matching, and sensitivity analyses were performed with differing matching ratios, observation periods, and PS stratifications. Statistical significance was set at *p* < 0.05.

## 5. Conclusions

In conclusion, PPI co-prescription for >4 weeks with clopidogrel was associated with hospitalization of recurrent stroke within 1 year of the initial diagnosis with stroke based on two different analyses. The risk showed an increasing trend parallel to the time from the beginning of PPI co-prescription and differed according to individual PPI types. However, the association with recurrent MI in the PPI co-prescription group did not show consistent results in the multiple analyses. Further comprehensive large-scale studies, including multiple races and ethnicities, are required.

## Figures and Tables

**Table 1:** Baseline characteristics between PPI co-prescription and non-prescription groups in patients with stroke and myocardial infarction *.

	Stroke			Myocardial Infarction		
Variables	PPI Co-Prescription Groups (*n* = 8201)	PPI Non-Prescription Group (*n* = 61,987)	*p*-Value	PPI Co-Prescription Group (*n* = 1216)	PPI Non-Prescription Group (*n* = 4751)	*p*-Value
Sex						
Women, *n*(%)	3742 (45.8)	25,662 (41.4)	<0.001	670 (55.1)	3043 (64.0)	<0.001
Age	67.3 ± 10.9	66.1 ± 11.8	<0.001	69.6 ± 10.2	68.1 ± 11.5	<0.001
Aspirin co-prescription	3094 (37.8)	22,810 (36.8)	0.069	982 (80.8)	3608 (75.9)	<0.001
Comorbidity						
Hypertension	6357 (77.7)	46,388 (74.8)	<0.001	1080 (58.1)	4103 (86.4)	0.023
Diabetes mellitus	4041 (49.4)	32,432 (52.3)	<0.001	707 (58.1)	2662 (56.0)	0.185
Dyslipidemia	5499 (67.3)	38,245 (61.7)	<0.001	917 (75.4)	3534 (74.4)	0.463
Smoking history						
Never smoker	2610 (31.9)	16,837 (27.1)	<0.001	341 (28.0)	1146 (24.1)	0.020
Ex-smoker	728 (8.9)	4643 (7.5)		106 (8.7)	389 (8.2)	
Current smoker	1137 (13.9)	8531 (13.8)		159 (13.1)	625 (13.2)	
BMI (kg/m^2^)	24.43 ± 3.09	24.39 ± 3.05	0.479	24.11 ± 3.1	24.41 ± 3.08	0.032
Total cholesterol level (mg/dL)	202.52 ± 42.01	204.96 ± 45.07	<0.001	205.94 ± 44.95	207.52 ± 53.37	0.460

* The applicated database is the Korean National Health Insurance Service database. PPI, proton pump inhibitor; BMI, body mass index.

**Table 2:** Relative risk for hospitalization with recurrent stroke or myocardial infarction during PPI exposure according to PPI duration (self-controlled case series analysis) *.

	Recurrent Stroke (*n* = 1448)		Recurrent MI (*n* = 208)	
	N (%)	Relative Risk (95% CI)	N (%)	Relative Risk (95% CI)
RR of PPIs exposed periods (PPIs co-prescribed periods only)				
PPIs unexposed	795 (54.9)	1 (reference)	113 (54.3)	1 (reference)
PPIs exposed	653 (45.1)	2.09 (1.83–2.38)	95 (45.7)	1.47 (1.02–2.11)
Time from beginning of PPI co-prescription				
0~2 weeks	254 (17.6)	1.76 (1.50–2.07)	32 (15.4)	1.30 (0.83–2.04)
2~4 weeks	187 (12.9)	2.02 (1.68–2.43)	16 (7.7)	0.95 (0.54–1.69)
4~6weeks	90 (6.2)	3.02 (2.36–3.86)	19 (9.1)	2.33 (1.32–4.13)
6~8 weeks	47 (3.2)	2.81 (2.02–3.92)	11 (5.3)	1.99 (0.98–4.03)
>8weeks	75 (5.2)	5.57 (4.06–7.64)	17 (8.2)	3.80 (1.93–7.45)
RR of PPIs exposed periods (included PPIs washout periods)				
Non-risk periods	594 (41.0)	1 (reference)	85 (40.9)	1 (reference)
Risk periods	854 (59.0)	2.47 (2.16–2.81)	123 (59.1)	1.87 (1.31–2.65)

* The applicated database is the Korean National Health Insurance Service database. MI, myocardial infarction; PPI, proton pump inhibitor; RR, relative risk.

**Table 3:** Relative risk for hospitalization with recurrent stroke or myocardial infarction during PPI exposure according to PPI type (self-controlled case series analysis) *.

Type of PPIs		Recurrent Stroke		Recurrent MI	
		Number of Events (%)	Relative Risk (95% CI)	Number of Events (%)	Relative Risk (95% CI)
Omeprazole	PPIs unexposed	83 (60.6)	1 (reference)	7 (77.8)	1 (reference)
	PPIs exposed	54 (39.4)	1.84 (1.18–2.86)	2 (22.2)	0.33 (0.47–2.34)
	Non-risk periods	64 (46.7)	1 (reference)	5 (55.6)	1 (reference)
	Risk periods including washout periods	73 (53.3)	2.03 (1.34–3.08)	4 (44.4)	1.04 (0.13–8.40)
Esomeprazole	PPIs unexposed	181 (48.5)	1 (reference)	29 (58.0)	1 (reference)
	PPIs exposed	192 (51.5)	2.75 (2.12–3.57)	21 (42.0)	0.89 (0.36–2.18)
	Non-risk periods	128 (34.3)	1 (reference)	23 (46.0)	1 (reference)
	Risk periods including washout periods	245 (65.7)	3.18 (2.45–4.11)	27 (54.0)	1.18 (0.52–2.65)
Pantoprazole	PPIs unexposed	213 (58.5)	1 (reference)	42 (51.2)	1 (reference)
	PPIs exposed	151 (41.5)	1.61 (1.21–2.13)	40 (48.8)	2.56 (1.46–4.50)
	Non-risk periods	175 (48.1)	1 (reference)	35 (42.7)	1 (reference)
	Risk periods including washout periods	189 (51.9)	1.80 (1.37–2.35)	47 (57.3)	2.53 (1.47–4.36)
Rabeprazole	PPIs unexposed	295 (60.0)	1 (reference)	39 (67.2)	1 (reference)
	PPIs exposed	197 (40.0)	1.88 (1.49–2.36)	19 (32.8)	1.11 (0.54–2.24)
	Non-risk periods	232 (47.2)	1 (reference)	34 (58.6)	1 (reference)
	Risk periods including washout periods	260 (52.8)	2.02 (1.62–2.52)	24 (41.4)	1.14 (0.54–1.98)
Lansoprazole	PPIs unexposed	121 (71.2)	1 (reference)	21 (65.6)	1 (reference)
	PPIs exposed	49 (28.8)	1.32 (0.87–2.01)	11 (34.4)	0.58 (0.22–1.52)
	Non-risk periods	99 (58.2)	1 (reference)	16 (50.0)	1 (reference)
	Risk periods including washout periods	71 (41.8)	1.63 (1.12–2.38)	16 (50.0)	0.87 (0.33–2.24)
Dexlansoprazole	PPIs unexposed	9 (75.0)	1 (reference)	0	1 (reference)
	PPIs exposed	3 (25.0)	1.08 (0.18–6.49)	2	NA
	Non-risk periods	6 (50.0)	1 (reference)	0	1 (reference)
	Risk periods including washout periods	6 (50.0)	3.65 (0.71–8.78)	2	NA

* The applicated database is the Korean National Health Insurance Service database. MI, myocardial infarction; PPI, proton pump inhibitor; NA, not applicable.

**Table 4:** Cox regression analysis for recurrent stroke and myocardial infarction during 1 year after PPI co-prescription (cohort study) *.

Outcome	Cohort	Patients, *n*	Observation, Person years	Events	Incidence Rate ^a^	HR (95% CI)	*p*-Value
Stroke	PPI	373	240	81	337.5	1.34 (1.01–1.76)	0.04
	Non-PPI	1051	740	189	255.2	Reference	
MI	PPI	179	133	23	171.7	1.42 (0.79–2.49)	0.23
	Non-PPI	439	336	43	127.8	Reference	

* The applicated database is the National Health Insurance Service Common Data Model. ^a^ Incidence rate expressed per 1000 person years. MI, myocardial infarction; PPI, proton pump inhibitor; HR, hazard ratio.

**Table 5:** Sensitivity analysis with different matching ratios, observation periods, and stratifications (cohort study) *.

Analysis	Observation Period	Stroke HR (95% CI)	MI HR (95% CI)
PS matching 1:4 (main analysis)	12 months	1.34 (1.01–1.76)	1.42 (0.79–2.49)
1:4	6 months	1.42 (1.05–1.90)	2.15 (1.10–4.13)
1:1	12 months	1.52 (1.06–2.20)	1.33 (0.69–2.65)
1:1	6 months	1.56 (1.07–2.30)	2.12 (0.95–5.21)
PS stratification	12 months	1.37 (1.08–1.73)	1.15 (0.71–1.81)
	6 months	1.43 (1.10–1.84)	1.69 (0.96–2.92)

* The applicated database is the National Health Insurance Service Common Data Model. MI, myocardial infarction; PS, propensity score; HR, hazard ratio

## Data Availability

Data are contained within the article.

## Supplementary Materials

The following supporting information can be downloaded at: https://www.mdpi.com/article/10.3390/ph16091213/s1.
