# Supplementary material for: Impact of Concomitant Use of Proton Pump Inhibitors and Clopidogrel on Recurrent Stroke and Myocardial Infarction
Source: Pharmaceuticals (Basel). 2023 Aug 28;16(9):1213. doi: 10.3390/ph16091213 (PMC10535402; doi:10.3390/ph16091213)
Supplement: Supplementary file 1 [file pharmaceuticals-16-01213-s001.zip › pharmaceuticals-2541887-supplementary.pdf]

Supplementary Table S1. Baseline characteristics between PPI group and non-PPI group in the analysis of stroke (cohort study)

Characteristic	Before PS adjustment			After PS adjustment		
	PPI (n=442)	Non-PPI (n=11,078)	SMD	PPI (n=373)	Non-PPI (n=1,051)	SMD
Age group						
45–49	2.3	3.9	−0.09	2.7	2.9	−0.01
50–54	3.8	7.2	−0.15	4.3	6.6	−0.1
55–59	9	9.4	−0.01	9.9	6.3	0.13
60–64	13.3	12.5	0.03	13.7	12	0.05
65–69	17	17.5	−0.02	16.4	19.8	−0.09
70–74	20.4	18.3	0.05	19.3	20.4	−0.03
75–79	17.6	14.9	0.08	17.7	18	−0.01
80–84	10.4	8.5	0.07	10.2	8.4	0.06
85–89	4.5	3.9	0.03	4.8	3.1	0.09
Gender: female	54.3	48.4	0.12	55	53	0.04
Medical history						
Acute respiratory disease	64.5	56.3	0.17	63.8	68.1	−0.09
Chronic liver disease	9	7.4	0.06	9.9	11.4	−0.05
Chronic obstructive lung disease	10.2	6.7	0.13	8.6	10.6	−0.07
Dementia	14.9	12.6	0.07	15	12.5	0.07
Depressive disorder	22.4	17.7	0.12	22	24.8	−0.07
Diabetes mellitus	52.3	41.3	0.22	52.3	49.4	0.06
Gastroesophageal reflux disease	38.5	9.9	0.71	35.9	40.6	−0.1
Gastrointestinal hemorrhage	14.9	3.9	0.39	11.8	12.8	−0.03
Hyperlipidemia	67	57.6	0.2	65.1	67.7	−0.05
Hypertensive disorder	77.8	78.3	−0.01	76.9	81.4	−0.11
Osteoarthritis	25.6	21.8	0.09	26.3	31	−0.1
Pneumonia	14.9	8.3	0.21	13.9	15.6	−0.05
Renal impairment	8.4	4.4	0.16	7.2	10.1	−0.1
Rheumatoid arthritis	8.4	5.8	0.1	8	8.4	−0.01
Urinary tract infectious disease	9.5	6.8	0.1	8.3	10.2	−0.06
Visual system disorder	46.8	43.9	0.06	45.8	50.9	−0.1
Atrial fibrillation	8.8	5.5	0.13	8.3	7.2	0.04
Coronary arteriosclerosis	6.6	3.7	0.13	6.4	5.3	0.05
Heart disease	58.1	43.5	0.3	55.8	57.8	−0.04

Heart failure	19.9	10.6	0.26	18.2	16.7	0.04
Ischemic heart disease	43.7	27.9	0.33	42.4	41.7	0.01
Peripheral vascular disease	49.5	39.2	0.21	49.6	49.2	0.01
Malignant neoplastic disease	10.4	6.5	0.14	8.8	11.6	−0.09
Medication use						
Agents acting on the renin-angiotensin system	57.5	49	0.17	56.6	56.1	0.01
Antibacterials for systemic use	78.3	69.3	0.21	77.5	79.8	−0.06
Antiepileptics	18.6	16.4	0.06	18	17.6	0.01
Antiinflammatory and antirheumatic products	75.6	67.2	0.19	76.7	77	−0.01
Antineoplastic agents	9.5	5	0.17	9.1	8.3	0.03
Beta blocking agents	45.5	36.4	0.19	44.5	46.1	−0.03
Calcium channel blockers	58.8	56.3	0.05	56.3	58.8	−0.05
Diuretics	55.9	43.1	0.26	53.4	53.7	−0.01
Drugs for acid related disorders	87.6	75.6	0.31	86.1	90.5	−0.14
Drugs for obstructive airway diseases	54.5	43.4	0.22	52.8	58.1	−0.11
Drugs used in diabetes	36.7	28.8	0.17	35.4	34	0.03
Opioids	65.4	51.1	0.29	63.3	70.2	−0.15
Psycholeptics	79.2	64.7	0.33	77.7	80.4	−0.06
Charlson index - Romano adaptation	6.9	5.4	0.48	6.8	6.9	−0.04

Values are presented as proportion (%). The covariates of proportion over 5% was presented. Abbreviations: PPI, proton pump inhibitor; PS, propensity score; SMD, standardized mean difference.

Supplementary Table S2. Baseline characteristics between PPI group and non-PPI group in the analysis of myocardial infarction (cohort study)

Characteristic	Before PS adjustment			After PS adjustment		
	PPI (n=245)	Non-PPI (n=3,933)	SMD	PPI (n=179)	Non-PPI (n=439)	SMD
Age group						
45–49	4.5	6.5	−0.09	3.4	5.8	−0.12
50–54	6.1	11.2	−0.18	6.1	9.1	−0.11
55–59	9.4	12.4	−0.1	10.6	8.1	0.09
60–64	9.8	13.9	−0.13	9.5	12.7	−0.1
65–69	18	14.9	0.08	20.1	18.3	0.05
70–74	18.4	14.3	0.11	16.2	14.9	0.04
75–79	17.6	10.7	0.2	16.8	12.4	0.12
80–84	6.9	6.1	0.03	6.7	6.6	0

85–89	4.9	3.5	0.07	6.1	4.1	0.1
90–94	<2.0	0.4	0.06	<2.8	1.3	−0.01
Gender: female	44.5	33.5	0.23	46.4	44.7	0.03
Medical history						
Acute respiratory disease	66.1	56.8	0.19	63.1	70.4	−0.16
Chronic liver disease	9	9.4	−0.02	9.5	12.5	−0.1
Chronic obstructive lung disease	13.5	9	0.14	12.3	12.8	−0.01
Dementia	8.6	4.5	0.16	8.4	5	0.13
Depressive disorder	15.5	10.6	0.15	16.2	15.3	0.03
Diabetes mellitus	45.3	45.2	0	43.6	49.5	−0.12
Gastroesophageal reflux disease	37.1	10.3	0.66	31.3	38.2	−0.14
Gastrointestinal hemorrhage	12.2	4.1	0.3	12.8	11.5	0.04
Hyperlipidemia	71	71.2	0	71.5	74.5	−0.07
Hypertensive disorder	79.6	80.6	−0.02	78.8	83.4	−0.12
Osteoarthritis	29	17.1	0.28	27.9	26.3	0.04
Pneumonia	13.9	10.4	0.11	13.4	17.5	−0.11
Renal impairment	8.6	7	0.06	7.8	9.1	−0.05
Rheumatoid arthritis	6.1	5.3	0.03	6.1	8.1	−0.08
Urinary tract infectious disease	7.3	6.7	0.03	7.8	10.6	−0.1
Visual system disorder	46.5	38.2	0.17	44.7	46.6	−0.04
Atrial fibrillation	9.4	8.1	0.04	11.2	8.1	0.1
Cerebrovascular disease	12.2	13	−0.02	11.7	16.4	−0.13
Heart disease	96.3	95.3	0.05	95.5	95.9	−0.02
Heart failure	31.8	20.9	0.25	30.7	26.2	0.1
Peripheral vascular disease	33.5	29.3	0.09	30.7	37	−0.13
Malignant neoplastic disease	10.2	7.1	0.11	10.1	12.1	−0.06
Medication use						
Agents acting on the renin-angiotensin system	80.4	74.3	0.15	78.8	76.1	0.06
Antibacterials for systemic use	75.9	70.5	0.12	72.6	80.4	−0.18
Antidepressants	23.7	15.9	0.2	22.3	22.7	−0.01
Antiepileptics	12.2	10.4	0.06	10.6	13.2	−0.08
Antiinflammatory and antirheumatic products	68.2	61	0.15	65.9	69.6	−0.08
Antineoplastic agents	7.3	3.9	0.15	7.3	7.8	−0.02
Beta blocking agents	71.8	72.4	−0.01	69.3	72.1	−0.06
Calcium channel blockers	58.4	52	0.13	57.5	58.5	−0.02
Diuretics	52.2	45.3	0.14	50.3	51.7	−0.03

Drugs for acid related disorders	86.5	76.9	0.25	85.5	90.4	−0.15
Drugs for obstructive airway diseases	52.7	44.5	0.16	52.5	54.4	−0.04
Drugs used in diabetes	30.2	30.5	−0.01	30.2	36.2	−0.13
Lipid modifying agents	79.2	69.2	0.23	78.2	76	0.05
Opioids	71	60.6	0.22	69.8	71	−0.02
Psycholeptics	69.8	65.6	0.09	65.4	70	−0.1
Charlson index - Romano adaptation	6.9	5.6	0.38	6.8	6.9	−0.04

Values are presented as proportion (%). The covariates of proportion over 5% was presented. Abbreviations: PPI, proton pump inhibitor; PS, propensity score; SMD, standardized mean difference.

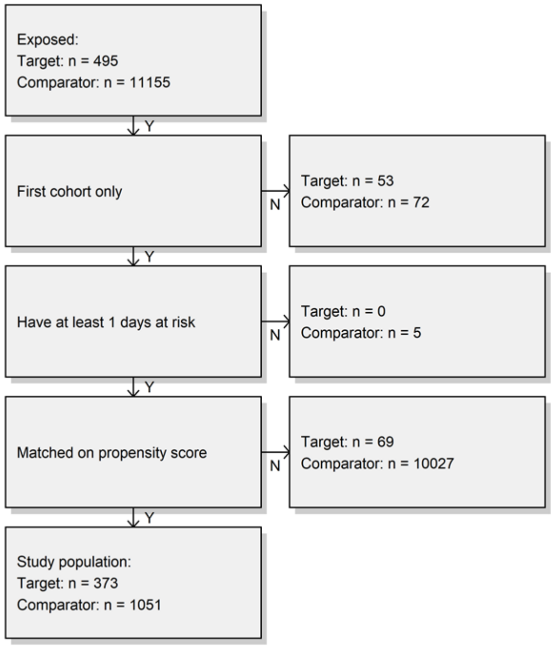

(A) Stroke

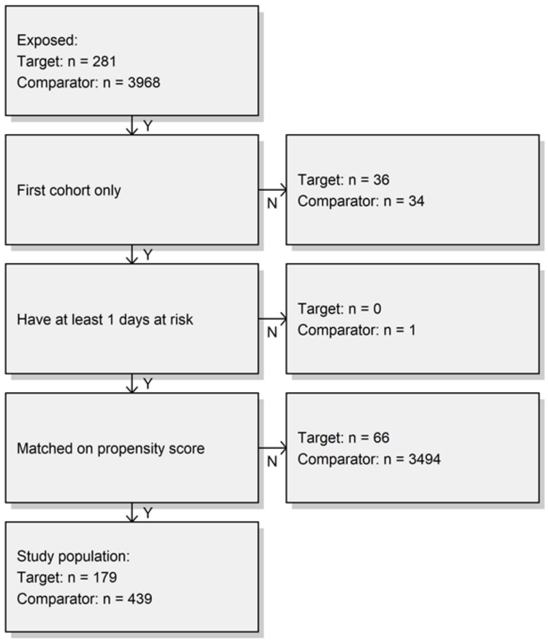

(B) Myocardial infarction

Supplementary Figure S1. Flow chart of cohort study in the analysis of stroke (A) and myocardial infarction (B).

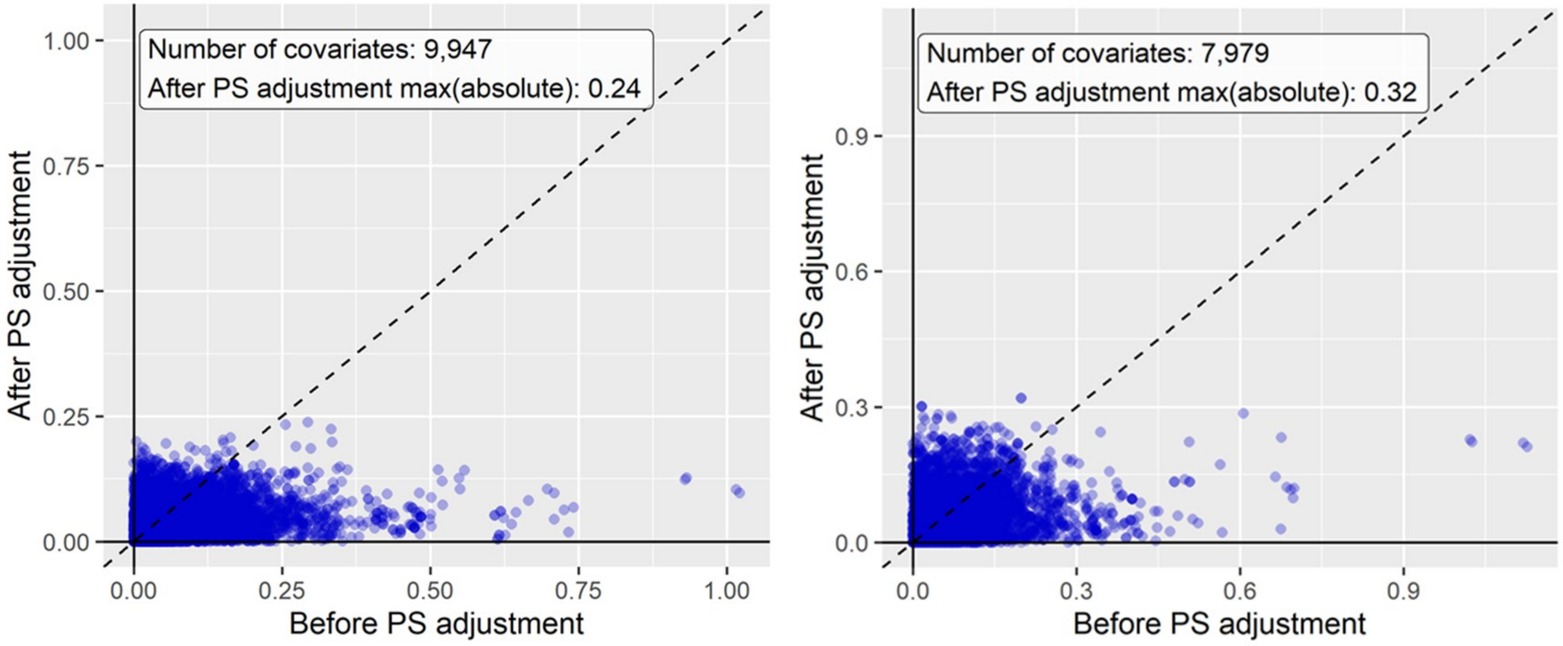

(A) Stroke

(B) Myocardial infarction

Supplementary Figure S2. Covariate balance before and after propensity matching in the cohort study.
Blue dots indicate the standardized mean difference of each covariate between the target and comparative cohorts before and after propensity-score matching.
PS, propensity score.
